# Serotonin transporter (5-HTT) gene network moderates the impact of prenatal maternal adversity on orbitofrontal cortical thickness in middle childhood

**DOI:** 10.1371/journal.pone.0287289

**Published:** 2023-06-15

**Authors:** Aleeza Sunderji, Heather D. Gallant, Alexander Hall, Andrew D. Davis, Irina Pokhvisneva, Michael J. Meaney, Patricia P. Silveira, Roberto B. Sassi, Geoffrey B. Hall

**Affiliations:** 1 Department of Psychology, Neuroscience & Behaviour, McMaster University, Hamilton, ON, Canada; 2 Department of Psychiatry, Faculty of Medicine and Health Sciences, McGill University, Montreal, QC, Canada; 3 Ludmer Centre for Neuroinformatics and Mental Health, Douglas Mental Health University Institute, McGill University, Montreal, QC, Canada; 4 Translational Neuroscience Program, Singapore Institute for Clinical Sciences and Brain–Body Initiative, Agency for Science, Technology and Research (A*STAR), Singapore Yong Loo Lin School of Medicine, National University of Singapore, Singapore, Singapore; 5 Department of Psychiatry, University of British Columbia, Vancouver, BC, Canada; Western University, CANADA

## Abstract

In utero, the developing brain is highly susceptible to the environment. For example, adverse maternal experiences during the prenatal period are associated with outcomes such as altered neurodevelopment and emotion dysregulation. Yet, the underlying biological mechanisms remain unclear. Here, we investigate whether the function of a network of genes co-expressed with the serotonin transporter in the amygdala moderates the impact of prenatal maternal adversity on the structure of the orbitofrontal cortex (OFC) in middle childhood and/or the degree of temperamental inhibition exhibited in toddlerhood. T1-weighted structural MRI scans were acquired from children aged 6–12 years. A cumulative maternal adversity score was used to conceptualize prenatal adversity and a co-expression based polygenic risk score (ePRS) was generated. Behavioural inhibition at 18 months was assessed using the Early Childhood Behaviour Questionnaire (ECBQ). Our results indicate that in the presence of a low functioning serotonin transporter gene network in the amygdala, higher levels of prenatal adversity are associated with greater right OFC thickness at 6–12 years old. The interaction also predicts temperamental inhibition at 18 months. Ultimately, we identified important biological processes and structural modifications that may underlie the link between early adversity and future deviations in cognitive, behavioural, and emotional development.

## Introduction

The prenatal period is one of the most rapid and formative stages of neurodevelopment. Foundational processes such as neurogenesis, synaptogenesis, migration, and differentiation are occurring to establish the baseline architecture for future development [1]. Importantly, such sensitive and dynamic periods of growth are typically characterized by a high degree of plasticity, rendering developing systems to be highly responsive and vulnerable to the environment [2, 3]. Therefore, it follows that prenatal development is strongly contingent upon environmental input provided in utero. These cues are taken to reflect the child’s expected postnatal conditions [4] and thereby function to tailor trajectories in ways that would be most conducive to survival after birth [3]. This idea of preparedness is captured in the long-standing fetal programming hypothesis, which posits that environmental insults during sensitive periods can exert lasting organizational effects on development [5]. In support, there is a wealth of research reporting alterations in structural, functional, cognitive, behavioral, and emotional development amongst children whose mothers experienced adverse events prenatally [6–16]. However, studies investigating the underlying mechanisms that give rise to such outcomes are needed.

There is a growing body of research which explores factors that alter an individual’s susceptibility to their environment. For instance, genetic variation can influence development so that individuals are better positioned to navigate within the conditions of their postnatal environment. In this way, functional allelic variations may be viewed as facilitating plasticity rather than predicting vulnerabilities [17]. Currently, there is a paucity of research which explores how genotype and one’s early environment interact to affect development during the instrumental middle childhood years. Accordingly, in the present study we aimed to address whether genetic variation in the serotonin transporter (5-HTT) gene network can moderate an individual’s vulnerability to the environment as early as the prenatal period during which environmental perturbations begin to affect long-term development.

Serotonin is one of the first neurotransmitters to be expressed during fetal development with serotonergic neurons detectable as early as 5 weeks gestation [18]. This neurotransmitter performs dual functions during development. In early periods, serotonin acts as a trophic factor regulating critical developmental processes such as cell division, differentiation, migration, myelination, synaptogenesis, and dendritic pruning [19]. In this role, 5-HT shapes the development of its own system and other closely related neural networks. During maturation, 5-HT functions as a modulatory neurotransmitter impacting arousal, stress responsivity as well as higher-order functions including cognition, attention, emotion, and learning [19]. It is important to note that while serotonin’s potential for neurotransmission does exist during early development, the mere presence of serotonergic synapses does not suggest that 5-HT is actively modulating neural activity in the fetal brain as it does in the mature/adult brain [20]. Furthermore, some 5-HT modulated developmental processes are not typically observed in the adult brain and occur exclusively during fetal and early postnatal periods. These processes include neuronal progenitor cell proliferation, neuronal migration, and axonal wiring [20]. Therefore, it is plausible that changes in serotonin signaling during these early critical periods of development can have substantial implications for long-term brain organization and function.

The serotonin transporter is the primary modulator of serotonergic activity (5-HT) in the brain. Alterations in its expression and availability influences the structural and functional development of neural systems, thereby subsequently affecting behaviour, emotion, and cognition [21]. Serotonergic activity has been broadly implicated in the etiology of a number of mood, anxiety, and neurodevelopmental disorders [22]. As such, it is important to understand the role of the serotonin transporter in regulating one’s vulnerability to environmental experiences. Caspi et al. (2003) found that a functional polymorphism in the promoter region of the serotonin transporter gene (SLC6A4) moderates the effect of stressful life events on the development of depression [23]. This seminal research sparked interest in exploring the moderating role the serotonin transporter gene polymorphism (5-HTTLPR) on the relation between early adversity and an increased risk for psychopathology, with a particular focus on a *potential* risk allele–the short allele. Carriers of the short allele show reduced transporter expression and function (e.g. reduced binding in limbic regions, decreased 5-HT reuptake into presynaptic neurons, etc), thereby suggesting a higher level of extracellular 5-HT [20]. Accumulating evidence has revealed that the short allele is associated with an increased risk for mood disorders, anxiety related temperaments, and structural and functional alterations in regions implicated in affective disorders [24–26].

However, attempts to replicate the interaction found by Caspi et al (2003) have not always been successful and some research has identified that not all of the outcomes associated with the short allele are negative [27]. It has been suggested that the inconsistent results stem from the single candidate gene approach used in these analyses. This approach considers single genes to be independently responsible for particular pathologies. However, complex, polygenic traits such as mental illnesses cannot solely be explained by a single gene and instead involve the interplay between multiple genes. It is more likely that weak effects from multiple genomic variations converge to influence a common biological system which in turn gives rise to instances of psychopathology [27, 28]. As such, polygenic risk scores (PRS) have shown some potential for clinical use in recent years. However, PRS fail to account for the biological events that underlie such complex states. Accordingly, a better approach should capture the biological processes involved in a particular disorder and provide us with a mechanistic link of how early adversity can lead to aberrant developmental outcomes. In the research reported here, we use a co-expression based polygenic risk score (ePRS) methodology developed by Silveira and colleagues to study how genetics interact with the prenatal environment to affect subsequent child development [28–30]. The ePRS is generated by identifying genes that are co-expressed with a particular gene of interest. The approach is based on the principle that genes which are expressed together, work together, and thereby comprise a gene network [28]. Here, we generate co-expression based polygenic risk scores that represent the function of the 5-HTT gene network in the amygdala expressed during prenatal development.

Cortical thickness (CT) has a well-defined developmental trajectory in middle childhood [31]. Specifically, the normative trajectory of CT development is characterized by a linear decline from early childhood to early adulthood [31, 32]. Therefore, gradual thinning reflects progressive maturation of brain organization, while reduced thinning (I.e. increased thickness) reflects a delay in cortical development [33, 34]. Factors such as synaptic pruning, dendritic development, changes in glial cell density, and myelination of white matter tracts can contribute to differences in CT [35–38]. Variations in CT are observed in various mental illnesses, thereby prompting investigation into potential risk factors that give rise to such alterations [39]. Some research has also reported regional differences in CT trajectories, however there is consistent evidence that OFC thickness shows a monotonic linear decrease with age [31, 32]. As such, our first aim is to explore whether adverse prenatal experiences and the 5-HTT gene network interact to predict cortical thickness of the orbitofrontal cortex (OFC) in middle childhood.

The OFC is the region of interest for several reasons. First, structural and functional alterations in regions of the Prefrontal Cortex have been observed following exposure to early life adversity, especially in regions actively involved in emotion regulatory processes [30, 40, 41]. The OFC, in particular, is highly interconnected with other brain regions responsible for sensory processing, emotion generation, emotion regulation, inhibitory control, executive control, and reward processing [42, 43]. It plays a critical role in integrating information from a variety of sources to guide behaviour. Therefore, in uncertain contexts, the OFC region is pivotal for generating adaptive and flexible behavioural responses [44]. Secondly, the rudimentary structure and connectivity of the prefrontal cortex is largely determined prenatally [45]. As such, environmental perturbations at this time can impact its gross architecture later in life [45, 46]. Moreover, during middle childhood, the development of the OFC undergoes continual refinement. As a pivotal region for the regulation of affect, cognition, and behaviour, its maturation also influences a child’s social and emotional development. Previous research has shown that structural changes in the OFC have been associated with poor behavioural and emotional outcomes [47, 48]. Therefore, it becomes important to understand the conditions under which alterations in OFC structure can ensue. In this study, we explore whether aberrant input (i.e. via altered 5-HTT-ePRS function) from a stress susceptible subcortical region influences the structural development of the OFC. Since the 5-HTT gene network is expressed in the amygdala, it would be reasonable to explore its influence on a closely connected region. Not only are both the amygdala and OFC highly regulated by serotonergic activity but animal research and a few human studies have revealed that they demonstrate strong bidirectional anatomical connectivity [49, 50]. fMRI research also indicates that the amygdala and both medial and lateral divisions of the OFC demonstrate functional connectivity [50, 51]. Furthermore, the degree of functional coupling between the OFC and amygdala is related to individual differences in emotion regulation and self-control [50, 52–54]. As such, we are interested in determining whether alterations in 5-HT activity within one region can moderate the structural developmental of another during middle childhood following exposure to prenatal maternal adversity.

Zhou and colleagues (2013) reported that cortical thickness asymmetry in frontal regions increases with age [55]. In the orbitofrontal region, rightward asymmetry (thicker right hemisphere relative to left) is detectable during middle childhood [55]. It is suggested that the maturation of cognitive functions may relate to the emerging hemispheric asymmetry from childhood into adulthood [55, 56]. Relatedly, a lack of asymmetry may be indicative of aberrant cognitive functioning or pathology [55, 56]. As such, given that a degree of asymmetry should be expected during middle childhood, we decided to explore hemispheric differences in the moderating role of the 5-HTT gene network by conducting separate analyses on the right and left OFC.

Given that the amygdala is a small, subcortical structure which is separated from other nearby regions by fine boundaries, manual tracing is considered to be the “gold-standard” volumetric segmentation technique. Commonly used automated segmentation tools are found to inflate estimates of amygdala volume, potentially reflecting poor boundary identification [57]. Particularly in pediatric samples, the agreement between manual and automated segmentation of the amygdala is said to be questionable [57]. Our concern regarding the accuracy of amygdala volume estimation in our sample of children guided our choice away from selecting the amygdala as the primary region of interest for this study. Further, when opting for an automated segmentation approach to conduct volumetric analyses of the amygdala, it is generally recommended that researchers acquire large sample sizes when expecting small effect sizes or exploring complex associations such as those involving the role of genetics [58].

The second aim of this study is to determine whether the 5-HTT gene network moderates the impact of prenatal adversity on early temperament. Behavioural inhibition (BI) can be defined as a "child’s initial behavioural reaction to unfamiliar people, objects, and contexts, or challenging situations’’ [59, 60]. This temperament can be detected as early as toddlerhood [26, 61]. Children that exhibit an inhibited temperament display withdrawn, fearful behaviour and increased vigilance in the face of novelty [62]. BI is an antecedent to shyness, which emerges in early childhood as social awareness begins to develop [63]. It has been suggested that a behaviourally inhibited temperament is also a precursor to anxiety and depression in late childhood and adolescence [62, 64–66]. Neurobiologically, BI is potentially attributed to high amygdala reactivity and poor emotion regulation ability by frontal regions [21, 60, 67]. As such, investigations into early indicators of poor regulatory ability are merited.

Ultimately, early temperament reflects a difference in reactivity to the environment which can be rooted in genetics and neurobiology [63]. However, much of the literature investigating differences in temperamental inhibition with relation to the 5HTTLPR polymorphism has led to inconsistent results [64, 68, 69]. Furthermore, prenatal stress is shown to impact the development of subcortical and cortical regions, and stress regulatory systems which underlie a behaviourally inhibited temperament [9, 70]. Interactions between an individual’s environment and the short 5-HTT allele have shown to increase the risk for BI in childhood [26]. For all these reasons, we wished to explore whether there is a 5-HTT gene network x prenatal environment interaction that predicts temperamental inhibition at 18 months.

Ultimately, this study aims to gain a better understanding of the biological mechanisms by which prenatal adversity can exert its downstream effects on temperament in toddlerhood (18 months) and neurodevelopment in middle childhood (6–12 years). We hypothesized that the impact of prenatal maternal adversity on OFC thickness at 6–12 years old and temperamental inhibition at 18 months will vary based on the function of the 5-HTT gene network in the amygdala.

## Materials and methods

### Sample

The Maternal Adversity, Vulnerability and Neurodevelopment (MAVAN) cohort is a community sample of Canadian mothers and their children who have been studied prospectively since the prenatal period. Dyads were recruited from two sites–either Hamilton, Ontario or Montreal, Quebec. The eligibility criteria entail mothers who are 18 years or older with singleton pregnancies and fluency in English or French. Severe maternal chronic illness, placental previa, a history of incompetent cervix, impending delivery, a fetus/infant affected by a major anomaly or born at a gestational age of <37 weeks or with birthweight less than 2000g comprise the exclusion criteria. The MAVAN project received approval from the ethics committee and affiliated university institutions (i.e. McMaster University, St. Joseph’s Healthcare Hamilton, McGill University, Université de Montréal, Royal Victoria Hospital, Jewish General Hospital, Centre Hospitalier de l’Université de Montréal, and Hôspital Maisonneuve-Rosemount). Informed written consent was obtained from all participants.

In order to study the presence of a gene network x environment interaction that moderates neurodevelopmental outcomes in middle childhood, MRI image acquisition was performed when MAVAN children reached 6–12 years of age (N = 90). Twenty-two structural scans were deemed unusable due to artifacts or missing files, and prenatal adversity data were not available for 11 subjects. Thus, the final sample consisted of 57 children aged 6–12 years old (boys: 30; girls: 27). The mean age of the sample was 9.28 years (SD = 1.46 years). Please refer to Table 1 for demographic information including maternal age at birth, gestational age, birth weight, duration of breastfeeding, smoking during pregnancy, maternal education, household income, maternal ethnicity and child ethnicity. For analyses that consider temperament at 18 months, an additional 5 participants were excluded (boys: 1, girls: 4) due to an incomplete data set (i.e. missing ECBQ scores at 18 months), thereby amounting to a total sample size of 52 children with a mean age of 9.28 years at scan (SD = 1.45, boys: 29, girls: 23).

**Table 1:** Sample demographics.

Characteristics	Total (n = 57)
Gender (boys)	52.6% (30)
Maternal age at birth (years)	30.36 (4.62)
Gestational age (weeks)	39.12 (1.18)
Birth weight (grams)	3259 (440)
Breastfed at least 3 months	70.9% (39)
Smoked during pregnancy	21.1% (12)
Maternal education (University Degree or above)	54.4% (31)
Low household income	13.7% (7)
Maternal ethnicity	
Non-Caucasian	10.5% (6)
Caucasian	89.5% (51)
Mixed Caucasian	0% (0)
Child ethnicity	
Non-Caucasian	7% (4)
Caucasian	84.2% (48)
Mixed Caucasian	8.8% (5)

### Early Childhood Behaviour Questionnaire (ECBQ)

The Early Childhood Behaviour Questionnaire (ECBQ) is a 201-item parental report measure that assesses 18 dimensions of temperament between ages 18 to 36 months [71]. It was developed to address the lack of detailed and comprehensive instruments available to study temperament during the period between infancy and childhood [71]. The ECBQ was administered to mothers when their child reached 18 months. Mothers were provided with frequently occurring situations and asked to indicate how often their child demonstrates a particular behaviour using a 7-point Likert scale (1 = never,7 = always, NA = child not observed in the given situation). Total scores on the 12-item shyness subscale were used as a proxy of behavioural inhibition during toddlerhood [72]. The ECBQ subscales are internally consistent, demonstrate good inter-rater reliability, and have moderate stability across time [71].

### Cumulative prenatal maternal adversity score

To obtain a measure of prenatal maternal adversity exposure, a cumulative score developed by Silveira et al (2017) was used [28]. This adversity score has been used across different cohorts and is consistently associated with poor physical and mental health outcomes [28, 73–77]. Each adverse event was coded by either its presence (1 point) or absence (0 points). For items represented with continuous scores, the 15^th^ or 85^th^ percentile was used as the cut-off for adding a point to the score. The total prenatal adversity score is the summation of all points. Instruments used to acquire information about various forms of adversity exposure were administered to women at 24–36 weeks of gestation and include the following:

The health and well-being questionnaire–a composite of several validated measures (short versions) [78]:

- A subscale from the daily hassles scale which measures the severity level (how often and to what degree) at which the woman lacked money for basic necessities such as food and electricity since the beginning of pregnancy. Responses were provided on a 3-point Likert scale (1 = somewhat severe, 2 = moderately severe, 3 = extremely severe) [79]. A score above 9 indicates a lack of money.
- The 9-item Marital Strain Scale of Pearlin and Schooler assesses chronic stress with a romantic partner. Women indicate how strongly they agree or disagree with various statements about non-acceptance, non-reciprocity, and frustration about role expectation using a 5-point Likert scale. Examples include (1) "I cannot completely be myself around my spouse." (2) "Generally, I give in more to my spouse’s wishes than he/she gives in to mine." [80]. A marital strain score greater than 2.9 suggests the presence of chronic stress with a romantic partner.
- The Abuse Assessment Screen consists of 5 items that assesses conjugal violence during pregnancy. Questions investigate the frequency, severity, perpetrator, and body sites of injury [81]. Indicating "yes" on questions 2, 3, and 4, indicate the presence of domestic violence or sexual abuse.
- The state version of Spielberger’s State-Trait Anxiety Inventory (STAI) assesses transient anxiety during pregnancy [82, 83]. Women are provided with 20 adjectives (e.g. "I feel tense") which are evaluated using a 4-point Likert scale (1 = not at all, 2 = somewhat, 3 = moderately, 4 = very much). An average score greater than 1.95 is suggestive of anxiety during pregnancy.
- Examination of the presence of current/resolved chronic disease during pregnancy (diabetes, hypertension, asthma) or severe acute conditions including vomiting, vaginal spotting or bleeding during the past 4–6 weeks, current anemia/constipation/blood in stool, and current vaginal/cervical/urinary tract infection.

Note the presence or absence of each of the above events were coded on the single item-level.

Maternal depressive symptomatology during pregnancy was evaluated using the Centre of Epidemiological Studies Depression Scale (CES-D) [84]. This 20-item self-report scale inquiries about the frequency of depressive symptoms in the past seven days allowing responses on a 3-point Likert scale (0 = never, 3 = most of the time). A score of 22 or above indicates the presence of prenatal depression. Smoking during pregnancy was coded as a binary outcome. Total household gross income below $30,000/year was scored as an adverse event. Information about birth weight and gestational age was attained from birthing units and the Canadian reference was used to calculate birth weight percentiles [85]. Birth weight percentile below the 10^th^ percentile or above the 90^th^ percentile in addition to a gestational age of 37 weeks or less were two additional instances of adversity.

### Cortical thickness

Structural MRI image acquisition took place when children were 6–12 years old. High-resolution T1-weighted MRI images were obtained using a GE MR750 Discovery 3T MRI scanner at the Imaging Research Centre at St. Joseph’s Healthcare (Hamilton, Canada) and a 3T trio Siemens scanner in the Cerebral Imaging Center at Douglas Mental Health Institute (Montreal, Canada). The following acquisition parameters were used: Hamilton– 3D inversion recovery-prepped, FSPGR (fast spoiled gradient-echo), axial acquisition, 512 x 512 matrix with 1mm slice thickness, FOV = 24cm, TE = 3200ms, TR = 10.308ms, flip angle = 9 degrees; Montreal– 1mm isotropic 3D MPRAGE (magnetization-prepared rapid acquisition with gradient-echo), sagittal acquisition, 256 x 256 matrix, TR = 2300ms, TE = 4ms, flip angle = 9 degrees. T1-weighted images were preprocessed using the recon-all processing pipeline in FreeSurfer V6.0 (https://surfer.nmr.mgh.harvard.edu). Steps included in this pipeline involve cortical surface reconstruction, motion correction, normalization, skull stripping, registration, subcortical segmentation, and cortical parcellation. Parcellations were based on the Desikan-Killiany atlas, and a manual quality check was performed on all images using VisualQC (https://raamana.github.io/visualqc) [86]. to ensure accuracy. Cortical thickness values were extracted using the aparcstats2table command and is defined as the distance between the pial surface and white matter boundary, measured in millimeters (mm).

### Genotyping

Buccal epithelial cells of children were genotyped for autosomal SNPs using genome-wide platforms PsychArray/PsychChip, Illumina. Quality control procedure was carried out using PLINK 1.951 [87, 88]. Samples with a call rate less than 90% were removed. SNPs that had a low call rate (<95%), low *p*-values on the Hardy-Weinberg Equilibrium (HWE) exact test (*p* < 1e-40), and minor allele frequency (MAF) < 5% were removed. After imputation using the Sanger Imputation Service [89] and the Haplotype Reference Consortium (HRC) as the reference panel (release 1.1), SNPs with an info score >0.80 were kept for analysis corresponding to 20,790,893 autosomal SNPs.

The population structure of the MAVAN cohort was described using principal component analysis, which was conducted on the genotyped autosomal SNPs with MAF > 5% with the following pruning parameters for linkage disequilibrium: not in high linkage disequilibrium (r^2^ > 0.2) across 50 kb regions and a sliding window of 5 SNPs [90, 91]. To account for population stratification, the first three PCs were included in all subsequent analyses.

### Serotonin transporter co-expression based polygenic risk score (5-HTT-ePRS)

The serotonin transporter co-expression based polygenic risk score (5-HTT-ePRS) was generated by identifying genes that are co-expressed with the serotonin transporter in the amygdala. Full details of the protocol can be found in Silveira et al. (2017), Miguel et al. (2019), and Dass et al (2019) [28–30]. The 5-HTT-ePRS was created using: GeneNetwork (http://genenetwork.org), BrainSpan (http://www.brainspan.org), NCBI Variation Viewer (https://www.ncbi.nlm.nih.gov/variation/view), and The Genotype-Tissue Expression (GTEx) (https://gtexportal.org/ home/). Using GeneNework a list of genes that are co-expressed with the 5-HTT within the amygdala in mice was prepared with a threshold of absolute value of r ≥ 0.5. Further, the list of mouse genes was converted to human orthologs by matching mouse EnsemblID with human EnsemblID in Mouse Genomic Informatics (MGI) database. The list was narrowed by selecting genes that are expressed in the human brain and which are 1.5 times more present during the prenatal period as compared to adulthood. SNPs that were functionally associated with gene expression were gathered using the National Center for Biotechnology Information, U.S. National Library of Medicine (NCBI Variation Viewer)—GRCh37.p13. Based on SNP GTEx data in the human amygdala, a final list of common SNPs was reached. Linkage disequilibrium clumping (r^2^ < 0.25) was performed to account for correlations between SNPs and resulted in 463 SNPs included in the 5-HTT-ePRS score. To calculate 5-HTT-ePRS score, the number of alleles (genotyping data) at each cis-SNP was weighted by the estimated effect from a regression model predicting gene expression from genotype. The direction of the correlation between genes and 5-HTT gene expression was also accounted for. Summation of these values over all SNPs comprises the 5-HTT-ePRS score.

### Statistical analysis

All statistical analyses were performed in R Studio (https://www.R-project.org/) [92]. Descriptive statistics for variables of interest can be found in Table 2. Student’s t-test for independent samples were conducted to detect group differences (sex, site) and Pearson’s correlational analyses allowed us to explore associations between four primary study variables–prenatal adversity, 5-HTT-ePRS score, cortical thickness values, and ECBQ shyness scores. Stepwise multiple linear regression analysis was performed to study the presence of a gene network x environment interaction that predicts an inhibited temperament at 18 months and cortical thickness at 6–12 years old. Predictor variables were the prenatal adversity score, genetic score (5-HTT-ePRS), and the interaction term. Response variables were either right mOFC thickness, left mOFC thickness, right lOFC thickness, left lOFC thickness, or the ECBQ shyness subscale scores. Models were adjusted for covariates including sex, age, and/or scanning site. The Akaike Information Criterion (AIC) was used to determine the best fitting linear model as it discourages overfitting by introducing a penalty as the number of parameters increases.

**Table 2:** Descriptive statistics.

	Mean ± SD	Range
**Age (years)**	9.28 ± 1.46	6.95–12.5
**5-HTT-ePRS**	0.003 ± 0.007	-0.013–0.015
**Prenatal adversity score**	2.3 ± 1.19	0–6
**BI 18m (ECBQ)**	3.052 ± 0.944	1.42–5.25
**Cortical thickness (mm)**		
**R mOFC thickness**	2.768 ± 0.182	2.319–3.247
**R lOFC thickness**	2.852 ± 0.133	2.617–3.135
**L mOFC thickness**	2.761 ± 0.166	2.412–3.286
**L lOFC thickness**	2.982 ± 0.152	2.671–3.374

Note: 5-HTT-ePRS = serotonin transporter co-expression-based gene network; BI = behavioural inhibition measured as 18 months using the early childhood behaviour questionnaire (ECBQ); R mOFC = right medial orbitofrontal cortex; R lOFC = right lateral orbitofrontal cortex; L mOFC = left medial orbitofrontal cortex; L lOFC = left lateral orbitofrontal cortex

## Results

When comparing if measures of cortical thickness differed depending on acquisition site, a borderline significant difference was found for right mOFC thickness values (t = -2.090, p = 0.047). However, after controlling for age and sex, the relationship was no longer significant (β = 0.109375, p = 0.149). No significant difference was detected in the level of maternal adversity exposure between Hamilton and Montreal samples. See Table 3.

**Table 3:** Sex and site differences in age, maternal adversity, 5-HTT-ePRS, behavioural inhibition and cortical thickness.

	Sex	Site
**Age (years)**	0.624 (0.535)	10.556 (p < 0.05) *
**Prenatal adversity**	-1.083 (0.284)	-0.882 (0.386)
**5-HTT-ePRS**	-0.791 (0.433)	0.448 (0.657)
**BI 18m (ECBQ)**	-0.430 (0.669)	-1.605 (0.118)
**Cortical thickness (mm)**		
**R mOFC thickness**	-1.204 (0.234)	-2.090 (0.047) *
**R lOFC thickness**	1.034 (0.306)	-1.763 (0.088)
**L mOFC thickness**	0.092 (0.927)	-1.098 (0.283)
**L lOFC thickness**	0.184 (0.855)	-1.282 (0.21)

Note: site = scanning site at which MRI scans were conducted (Hamilton or Montreal); prenatal adversity = continuous prenatal adversity score; 5-HTT-ePRS = serotonin transporter co-expression-based gene network; BI = behavioural inhibition measured at 18 months using the early childhood behaviour questionnaire (ECBQ); R mOFC = right medial orbitofrontal cortex; R lOFC = right lateral orbitofrontal cortex; L mOFC = left medial orbitofrontal cortex; L lOFC = left lateral orbitofrontal cortex. Significant p-value **(p<0.05)**.

A moderate, negative correlation was observed between the left lOFC and age (r = -0.320, p = 0.015). BI during toddlerhood was moderately correlated with right lOFC (r = 0.306), and left medial (r = 0.298) and lateral (r = 0.315) OFC thickness values at 6–12 years old. Increased behavioral inhibition displayed at 18 months was associated with greater cortical thickness in regions of the OFC during middle childhood. Prenatal maternal adversity was not associated with 5-HTT-ePRS score, ECBQ subscale scores at 18 months, nor OFC thickness at 6–12 years old. The 5-HTT-ePRS was not correlated with a BI at 18 months. See Table 4.

**Table 4:** Correlations between variables related to maternal adversity.

	Prenatal adversity	5-HTT-ePRS		BI 18m (ECBQ)	Age (years)
**Prenatal adversity**		0.210 (0.116)		-.182 (0.197)	0.140 (0.300)
**BI 18m (ECBQ)**	-.182 (0.197)	0.141 (0.317)			
**Cortical thickness (mm)**					
**R mOFC thickness**	0.236 (0.077)		0.095 (0.484)	0.197 (0.161)	-0.255 (0.056)
**R lOFC thickness**	0.091 (0.500)	-0.004 (0.975)		**0.306 (0.027)**	-0.267 (0.045)
**L mOFC thickness**	-0.043 (0.749)	-0.040 (0.766)		**0.298 (0.032)**	-0.260 (0.051)
**L lOFC thickness**	0.231 (0.084)	0.007 (0.960)		**0.315 (0.023)**	**-0.320 (0.015)**

Note: prenatal adversity = continuous prenatal adversity score; 5-HTT-ePRS = serotonin transporter co-expression-based polygenic risk score; BI = behavioural inhibition measured at 18 months using the early childhood behaviour questionnaire (ECBQ); R mOFC = right medial orbitofrontal cortex; R lOFC = right lateral orbitofrontal cortex; L mOFC = left medial orbitofrontal cortex; L lOFC = left lateral orbitofrontal cortex. Significant p-value **(p<0.05)**

### Multiple linear regression analysis

#### G x E interaction predicting right mOFC and lOFC thickness in middle childhood

For our multiple linear regression analysis with right mOFC thickness as the outcome variable, the best fitting model included the main effect of prenatal adversity, the main effect of the 5-HTT-ePRS, their interaction, and MRI scanning site (either Hamilton or Montreal) as a covariate (see Table 5). This model explained nearly 15% of the variance in right mOFC thickness (adj R^2^ = 0.1499, p = 0.014). Similarly, right lOFC thickness was best modelled as a function of prenatal adversity, 5-HTT-ePRS, their interaction, after adjusting for age and sex (see Table 6). Approximately 15% of the variation in thickness was explained by the aforementioned linear model (adj R^2^ = 0.1476; p = 0.021). The proportion of variance accounted for by the main effect of prenatal adversity, 5-HTT-ePRS, and their interaction–beyond the effects of covariates, please see Table 7.

**Table 5:** Multiple linear regression analysis results–prenatal adversity x 5-HTT-ePRS interaction model predicts right medial orbitofrontal cortex thickness.

	unstandardized		standardized					
	*β*	SE	*β*	*SE*	p-value	R^2^	Adjusted R^2^	p
						0.2106	0.1499	**0.014**
intercept	2.613	0.055	0.069	0.126	0.588			
site	0.133	0.049	0.346	0.127	**0.009**			
prenatal	0.052	0.023	0.202	0.128	0.120			
5-HTT-ePRS	18.301	8.489	0.048	0.127	0.708			
prenatal:5-HTT-ePRS	-7.410	3.535	-0.334	0.159	**0.041**			

Note: β = beta coefficient (slope); SE = standard error; site = location at which MRI scanning was conducted (either Hamilton or Montreal); prenatal = continuous prenatal adversity score; 5-HTT-ePRS = serotonin transporter co-expression-based polygenic risk score

**Table 6:** Multiple linear regression analysis results–prenatal adversity x 5-HTT-ePRS interaction model predicts right lateral orbitofrontal cortex thickness.

	unstandardized		standardized					
	*β*	*SE*	*β*	*SE*	p-value	R2	Adjusted R2	p
						0.2237	0.1476	**0.021**
intercept	3.140	0.112	0.088	0.127	0.493			
age	-0.037	0.012	-0.411	0.133	**0.003**			
sex	-0.063	0.034	-0.238	0.127	0.067			
prenatal	0.043	0.017	0.210	0.130	0.113			
5-HTT-ePRS	16.085	6.480	0.017	0.128	0.895			
prenatal:5-HTT-ePRS	-6.856	2.670	-0.424	0.165	**0.013**			

Note: β = beta coefficient (slope); SE = standard error; prenatal = continuous prenatal adversity score; 5-HTT-ePRS = serotonin transporter co-expression-based polygenic risk score

**Table 7:** Proportion of variance accounted for by the main effect of prenatal adversity, 5-HTT-ePRS, and their interaction (no covariates).

Outcome variable	R^2^	Adjusted R^2^	p-value
R mOFC thickness	0.098	0.047	0.138
L mOFC thickness	0.004	-0.052	0.975
R lOFC thickness	0.048	-0.006	0.456
L lOFC thickness	0.058	0.005	0.358
BI at 18 months	0.126	0.071	0.089

Note: R mOFC = right medial orbitofrontal cortex; L mOFC = left medial orbitofrontal cortex; R lOFC = right lateral orbitofrontal cortex; L lOFC = left lateral orbitofrontal cortex; BI = behavioural inhibition measured using the Early Childhood Behaviour Questionnaire (ECBQ)

**Fig 1:**
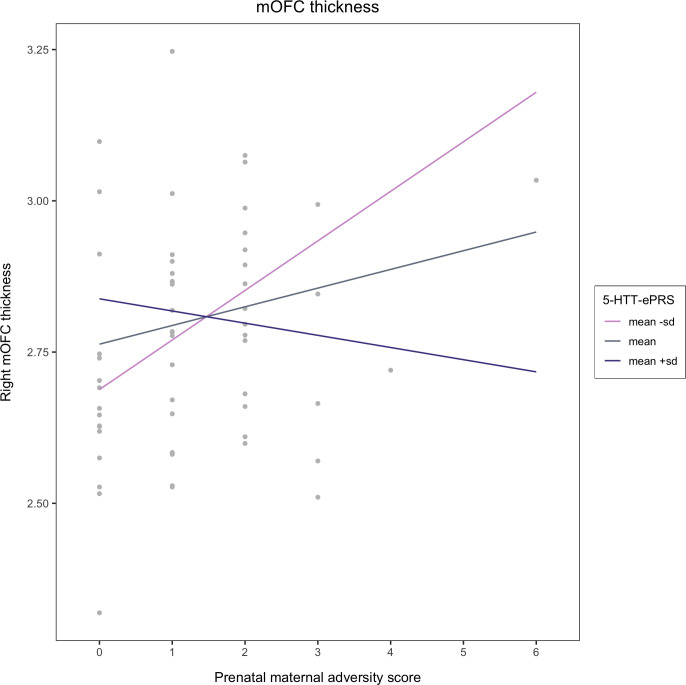
Prenatal maternal adversity and 5-HTT-ePRS prediction of right medial OFC thickness. In the presence of a low functioning co-expression based serotonin transporter gene network (mean -SD), there is a positive association between prenatal adversity exposure and right medial orbitofrontal cortex thickness (i.e. higher levels of prenatal adversity predict higher right medial orbitofrontal cortex thickness values).

**Fig 2:**
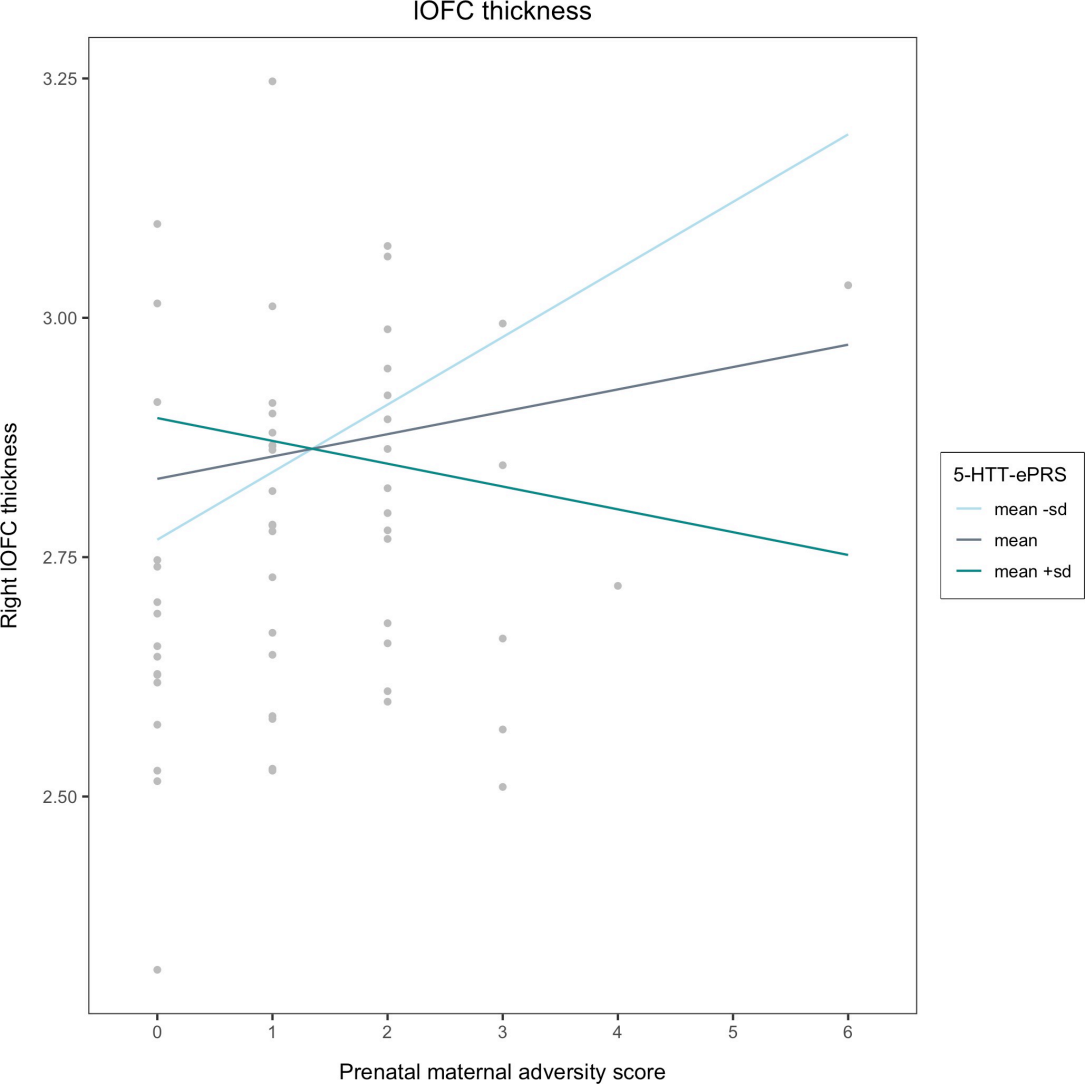
Prenatal maternal adversity and 5-HTT-ePRS prediction of right lateral OFC thickness. In the presence of a low functioning co-expression based serotonin transporter gene network (mean -SD), there is a positive association between prenatal adversity exposure and right lateral orbitofrontal cortex thickness (i.e. higher levels of prenatal adversity predict higher right lateral orbitofrontal cortex thickness values).

Ultimately, results from the multiple linear regression analyses revealed a significant interaction between prenatal adversity and the 5-HTT gene network that predicts both right medial and lateral orbitofrontal cortex thickness in middle childhood (medial: β = -0.334; p = 0.041; lateral: β = -0.424, p = 0.013). To probe the exact directionality of these interactions, a post-hoc simple slope analysis was conducted. 5-HTT-ePRS scores were held at the mean, 1 SD above the mean, and 1 SD below the mean. We found that at low 5-HTT-ePRS scores (mean -1SD), higher levels of prenatal adversity exposure are associated with greater right medial (p = 0.014) and lateral (p = 0.007) orbital frontal cortices at 6–12 years old (See Figs 1 and 2).

### G x E interaction predicting left mOFC and lOFC thickness in middle childhood

No significant interaction between adversity exposure and the 5-HTT gene network that could predict left mOFC (covariates = age) or left lOFC (covariates = age and site) thickness at 6–12 years old (medial: β = -0.145, p = 0.415; lateral: β = -0.228, p = 0.162) was detected.

### G x E interaction predicting BI at 18 months

The interaction between prenatal adversity and the 5-HTT gene network was significantly associated with BI at 18 months (β = 0.35, p = 0.034). However, our full model including the main effect of prenatal adversity, the 5-HTT-ePRS score, and their interaction did not reach statistical significance (adjusted R^2^ = 0.071, p = 0.089).

### Exploratory analysis: BI at 18 months and G x E interaction predicting CT at 6–12 years

As an exploratory analysis, we decided to investigate whether the degree of BI displayed at 18 months and the gene network x environment interaction can predict cortical thickness of the OFC in middle childhood. Our multiple linear regression analysis showed that after controlling for sex and scanning site, there was a main effect of temperament (β = 0.050, p = 0.009) on right lOFC thickness at 6–12 years old and a significant interaction (β = -7.505, p = 0.005). Together, both the gene (5-HTT-ePRS) x environment (prenatal adversity) interaction and BI at 18 months predict around 24% of the variation in right lOFC thickness in middle childhood (adj R^2^ = 0.244, p = 0.004).

## Discussion

We report that the function of the 5-HTT gene network in the amygdala moderates the impact of prenatal adversity on structural neurodevelopment in middle childhood. Specifically, in the presence of a low functioning 5-HTT gene network, there is a positive correlation between prenatal maternal adversity exposure and cortical thickness in both the right medial and lateral orbitofrontal cortices at ages 6–12 years old. The 5-HTT gene network x prenatal adversity interaction, however, did not effectively capture variation in the degree of BI exhibited during toddlerhood.

Our findings build upon previous research which has shown that the expression of 5-HTT, a primary modulator of 5-HT activity, impacts the structure and connectivity of neural circuitry (i.e. amygdala, anterior cingulate, and prefrontal regions) involved in the pathophysiology of affective disorders [18, 21]. Similarly, another line of evidence identifies that atypical neural migration and cortical development is observed upon manipulating 5-HT activity pharmacologically. Specifically, in utero exposure to SSRIs (i.e. increased 5-HT activity) has been associated with low levels of reelin, a protein important for correct neural migration [93]. Since reelin typically decreases with gestational age [93], low levels may be indicative of accelerated neurodevelopment. The above findings suggest that distinct developmental stages/processes can be disrupted depending on the period at which 5-HT availability is altered. It is thus important to attend to the developmental period at which 5-HT availability is modified, how and what factors contribute to such alterations (e.g. genetics, environment, GxE interactions, epigenetics), as well as the downstream outcomes of early signaling interruptions. Here, we report that alterations in 5-HT signaling in the amygdala during the prenatal period can affect right orbitofrontal cortical thickness development in middle childhood.

Given the recognized association between psychopathology and both 5-HT signaling and prenatal adversity exposure, it is interesting to observe an interaction between the 5-HTT gene network and prenatal maternal adversity that predicts atypical development in a region central to emotion processing and regulation. In particular, we found that a low functioning 5-HTT gene network in the amygdala moderates the impact of adverse prenatal experiences on the development of the right medial and lateral OFC such that these regions present greater thickness at higher levels of adversity 6–12 years after exposure. Our results may suggest that the OFC development may be one neural target of prenatal adversity, but the effect can be influenced by the development and communication with nearby regions. From a neurobiological standpoint, the amygdala and OFC exhibit bidirectional structural connectivity, functional connectivity, and are profoundly modulated by 5-HT signalling as they receive dense serotonergic projections [94, 95]. Both regions are also implicated in a network associated with generating and regulating emotional responses [94]. The amygdala signals information about emotional salience of environmental stimuli to which the OFC exerts top-down modulation to regulate one’s behavioural response. Decision-making, behavioural flexibility, reward processing, inhibition, sensory integration, and attention all require OFC involvement [42, 96]. Thus, it is not surprising that morphometric and functional alterations in the amygdala and OFC are putative neural correlates of many emotional and behavioural problems. For example, thinning in the OFC is linked to a variety of psychiatric conditions such as depression, anxiety, ADHD, as well as internalizing and externalizing symptoms [8, 34, 47, 97–99]. Some research has also found that cortical thickness in the vmPFC is positively associated with hemodynamic response in subcortical regions during adulthood [35], suggesting that a thinner vmPFC may be indicative of a decreased ability to regulate negative affective states. Conversely, Whittle et al., (2020) found that increases in internalizing symptoms across ages 8–10 years was associated with reduced thinning in the bilateral OFC [34]. Behaviourally, fear extinction is positively related to the thickness of the right vmPFC [100]. Together, these research findings suggest that regional differences in cortical thickness are linked to variations in certain cognitive processes and emotion regulation ability, yet there still exists discrepancies in the directionality of change. Our results add to this pool of knowledge by demonstrating that the impact of higher levels of prenatal maternal adversity on OFC thickness is moderated by functional alterations of the 5-HTT gene network within a close connected region (i.e. amygdala). Based on previous literature, such developmental change may ultimately have implications for future behavioural and emotional development.

Our results revealed a positive relationship between prenatal adversity and right OFC thickness in the presence of a low functioning 5-HTT gene network. It may be plausible that a low functioning 5-HTT gene network reflects an underlying network of genes involved in biological processes that interfere/delay typical CT maturation in middle childhood with heightened exposure to prenatal maternal adversity. Differences in maturational trajectories have been noted in the literature. For instance, the stress acceleration hypothesis posits that exposure to early life adversity promotes accelerated maturation of emotion circuits [40]. However, much evidence in support this theory stems from a body of research reporting that caregiver deprivation is associated with accelerated maturation in mPFC-amygdala circuitry [101, 102]. Specifically, negative functional connectivity between these regions is reached at an earlier timepoint [102]. It is also suggested that accelerated maturation of some circuits may come at the cost of delayed maturation in others [103]. Further studies are required to confirm whether a pattern of accelerated development upon ELA exposure extends to various structural metrics as well, if there is regional specificity, and whether acceleration is observed across various types of early adversity exposure. Multimodal imaging techniques can help elucidate whether there is congruency between structural and functional development. The findings from our study offer evidence of divergent structural maturation in the right OFC with higher levels of prenatal maternal adversity exposure and lower functioning of the 5-HTT gene network. Importantly, the unilateral hemispheric findings do corroborate with other research findings that suggest a pattern of right laterality in structural brain development upon early life stress exposure [104, 105]. Furthermore, the right prefrontal cortex is typically associated with inhibitory control [106]. Greater involvement of the right PFC is observed during tasks involving response selection and inhibition [107]. Likewise, suboptimal response inhibition in childhood is related to insufficient recruitment of the right VLPFC [107]. Evidence of PFC lateralization is also observed in psychopathologies. In particular, the right medial OFC is thinner in individuals with MDD compared to controls [108]. Similarity, a slower rate of cortical thinning in right frontal regions is associated with higher levels of inattention and hyperactivity/impulsivity during childhood [109]. Taken together, atypical right OFC development may represent a neurodevelopmental outcome of prenatal adversity exposure that could underpin aberrant inhibitory functioning. The 5-HTT-ePRS may moderate this effect of prenatal experiences on OFC thickness by influencing the progression of myelination and synaptic pruning.

The current study moves past the single-gene reductionist approach and acknowledges that genes do not code for disease states but rather, critical biological processes. Ultimately, while the 5-HTT has received much research attention as the primary regulator of 5-HT activity, it does work among a complex network of genes, transporters, receptors, their subtypes, and metabolic enzymes which collectively determine overall 5-HT signaling efficiency [19, 20, 28, 73]. It is possible that weak effects from multiple genes as opposed to a single gene polymorphism underlie atypical cognitive, emotional, behavioral, and neurodevelopmental outcomes [28]. Therefore, the gene network approach may serve utility in rectifying the discordant findings from methylation patterns and outcome associations with single genes. Instead, the gene network models multiple, critical *biological processes* that contribute long-term programming of early adverse experiences. Specifically, the 5-HTT gene network consists of genes involved in epigenetic modifications (e.g. DNA methylation, histone phosphorylation and methylation, chromatic remodeling) and other cellular and metabolic processes [73]. Ten of 35 genes in the network are identified as epifactors which could play a role in epigenetic processes that program the trajectory of OFC thickness development [73]. An enrichment analysis also revealed that the 5-HTT-ePRS network is highly involved in biological processes underlying nervous system development, and muscarinic acetylcholine and nicotine receptor signaling pathways [73]. Ultimately, the 5-HTT-gene network in the amygdala may be involved in biological processes that facilitate the biological embedding of adversity.

We also found that the interaction between prenatal adversity x 5-HTT-ePRS is significantly related to temperamental inhibition at 18 months. This finding indicates that there may be value in considering the moderating role of co-expression-based gene networks when studying the relationship between prenatal maternal adversity and behavioural/developmental outcomes [28]. However, our overall model including the main effects of prenatal adversity, the 5-HTT-ePRS, and the 5-HTT-ePRS x prenatal adversity interaction was not able to effectively capture differences in BI at 18 months given by a low and insignificant amount of variance explained by the predictor variables. Further, due to our small sample size, it is unclear whether the relationship between the interaction and BI will generalize to other samples. Future work should increase sample size and consider additional variables that are hypothesized to influence the degree of BI exhibited in toddlerhood. Such analyses may be better able to detect a stronger and more stable relationship between the interaction and BI at 18 months and better capture the variation. Nonetheless, our results suggest that it could be useful to consider the effect of gene network x environment interactions when studying early infant behaviour.

Our exploratory analysis revealed that the interaction between prenatal adversity and the 5-HTT gene network in addition to a main effect of BI at 18 months predicts almost a quarter (24%) of the variation in right lOFC thickness in middle childhood. The amygdala and OFC can be thought of as a network of structures subserving a behavioural spectrum ranging from disinhibition and impulsiveness to an inhibited and cautious temperament [70]. It is estimated that approximately 15–20% of children are born with extreme BI and this temperament exhibits moderate stability to adulthood [110–113]. Previous research efforts have found evidence of early temperament affecting structural development of the OFC. For instance, Hill et al (2010) note that 5-year-old children who demonstrated a heightened reaction when encountering a new child had greater right to left OFC volume ratios [70]. Such results suggest that the anatomical characteristics of the OFC may indicate a disposition towards either an inhibited or impulsive temperament. The present study extends upon this research by offering evidence that the degree of BI displayed in toddlerhood (18 months) can predict right lOFC thickness in childhood in conjunction with a 5-HTT-gene network x prenatal environment interaction. Similar results were found by Schwartz et al (2010) who observed that temperamental differences at 4 months predicted differences in left orbital and right ventromedial PFC thickness at 18 years old [114]. Hill et al (2010) also reported that BI measured at 5 years old was associated with greater right OFC volume at 15 years old [70]. It is therefore possible that OFC morphology may be one neural substrate that facilitates long-term maintenance of this temperament.

## Limitations and future directions

Our research does not go without limitations and an opportunity for future work. Firstly, the results of our study are limited by small sample sizes (power ranging from 0.62–0.67) and the lack of correction for multiple comparisons. It is possible that the reported effect sizes may be inflated due to sampling bias. For these reasons, we encourage replication of this research in an independent cohort with a larger sample size.

Future studies should also account for continued exposure to adversity after birth. Understanding the balance between shared and independent variance explained by prenatal and postnatal environments would further our understanding of the conditions under which particular long-term developmental consequences ensue.

Furthermore, assessing the role of OFC thickness in mental health is needed to elucidate whether delayed maturation is protective against or poses risk for poor cognitive, behavioural, and emotional development. Currently, there is a dearth of research that explores relations between CT and pediatric and adolescent mental health [37]. Since it is reported that 50% of all mental illnesses are present by age 15 years yet remain undetected [115], identifying early indicators of psychopathology can bring us one step closer towards designing appropriately targeted and timed interventions.

Multimodal, longitudinal analyses capturing development into adulthood are also required to confirm whether the observed developmental trajectory in middle childhood leads to atypical outcomes at a later time point or whether we simply identified a temporary deviation along a normative path. In the latter case, we may expect to see differences in the rate of maturation during one developmental period followed by a return to average development further into adulthood.

Finally, since it is possible to observe accelerated maturation in some regions/networks at the cost of delayed maturation in others [103], whole brain multimodal investigations would assist in furthering our knowledge about whether ELA is associated with patterns of compensatory development.

## Conclusion

Our research revealed that an interaction between the 5-HTT gene network expressed in the amygdala and prenatal maternal adversity predicts structural neurodevelopment in middle childhood. The gene network approach offers insight into potential biological processes that could lead to long-term programming of early adversity. We found that higher levels of prenatal maternal adversity exposure are associated with greater right OFC thickness values during middle childhood in the presence of a low functioning 5-HTT gene network. Future work should explore whether these cross-sectional results translate into longitudinal changes in OFC maturational trajectories. Interestingly, 5-HT signalling in the amygdala was associated with the structural development of its emotion regulatory counterpart, which helps us flexibly adapt our behaviour within changing situations. Through an exploratory analysis we also identified that temperamental inhibition at 18 months predicts right lateral OFC thickness at age 6–12 years. Together, both early temperament as well as the biological embedding of prenatal maternal adversity via the 5-HTT gene network may establish a developmental profile that programs an individual’s susceptibility to their environment.
